# Relapsed/Refractory Diffuse Large B-Cell Lymphoma: A Look at the Approved and Emerging Therapies

**DOI:** 10.3390/jpm11121345

**Published:** 2021-12-10

**Authors:** Yazeed Sawalha

**Affiliations:** Department of Internal Medicine, Division of Hematology, Arthur G. James Comprehensive Cancer Center, The Ohio State University Wexner Medical Center, Columbus, OH 43210, USA; Yazeed.sawalha@osumc.edu

**Keywords:** DLBCL, novel, emerging, FDA-approved

## Abstract

Approximately 40% of patients with diffuse large B cell lymphoma (DLBCL) do not respond or develop relapsed disease after first-line chemoimmunotherapy. A minority of these patients can be cured with autologous hematopoietic stem cell transplantation (AHCT). Although chimeric antigen receptor (CAR) T cells have transformed the treatment paradigm of relapsed/refractory DLBCL, only 30–40% of patients achieve durable remissions. In addition, many patients with relapsed/refractory DLBCL are ineligible to receive treatment with CAR T cells due to comorbidities or logistical limitations. Since 2019, the following four non-CAR T-cell treatments have been approved in relapsed/refractory DLBCL: polatuzumab in combination with bendamustine and rituximab, selinexor, tafasitamab plus lenalidomide, and loncastuximab. In this article, I review the data behind these four approvals and discuss important considerations on their use in clinical practice. I also review emerging therapies that have shown promising early results in relapsed/refractory DLBCL including the bispecific antibodies, antibody–drug conjugates, Bruton tyrosine kinase inhibitors, BCL2 inhibitors, immune checkpoint inhibitors, and epigenetic modifiers.

## 1. Introduction

Diffuse large B cell lymphoma (DLBCL) is the most common type of lymphoma in the United States, comprising approximately 20% of all lymphoid malignancies [1]. DLBCL is a biologically heterogeneous lymphoma with the following three subtypes based on cell-of-origin (COO) identification using gene expression profiling (GEP): germinal center B-cell (GCB), activated B-cell (ABC), and a third unclassified subtype [2]. As GEP is not widely available, several diagnostic algorithms based on immunohistochemistry (IHC) have been used to determine the COO, of which the most commonly used is the Hans algorithm, which differentiates GCB from non-GCB (ABC + unclassified subtypes) [3]. These subtypes not only constitute different molecular entities but may also respond differently to certain treatments, as discussed throughout the text. Another important consideration is the presence of genetic rearrangements of the *MYC*, *BCL2*, and *BCL6* genes that identifies patients with what is called double- or triple-hit lymphomas (*MYC* with *BCL2* and/or *BCL6* rearrangements). Double- and triple-hit lymphomas are now defined as “high-grade B-cell lymphoma (HGBCL) with rearrangements of *MYC* and *BCL2* and/or *BCL6*”, an entity separate from DLBCL in the 2016 revision of the World Health Organization lymphoma classification. HGBCL is associated with more aggressive clinical behavior and inferior outcomes [4]. 

Although most patients with DLBCL respond to first-line chemoimmunotherapy with R-CHOP (rituximab, cyclophosphamide, doxorubicin, vincristine, prednisone) or a similar regimen, approximately 40% of patients will have relapsed or refractory disease, most of whom will die from their lymphoma [5]. For more than two decades, the standard-of-care treatment for eligible patients with relapsed/refractory DLBCL has been second-line chemoimmunotherapy followed by consolidative autologous hematopoietic stem cell transplantation (AHCT). However, only a minority of patients with relapsed/refractory DLBCL can be cured with AHCT, as the majority are ineligible for AHCT due to age/comorbidities or having disease refractory to second-line chemoimmunotherapy. In addition, the long-term disease-free survival of patients who undergo consolidative AHCT is only about 50% [6,7]. Until recently, the treatment options for the patients who are ineligible for AHCT or those whose lymphoma relapsed after AHCT were limited mainly to chemoimmunotherapy regimens such as R-GemOx (rituximab, gemcitabine, oxaliplatin), BR (bendamustine, rituximab), or pixantrone, or the off-label use of ibrutinib or lenalidomide; all are used with palliative intent given their limited efficacy [8,9,10,11,12,13]. Pixantrone, which was approved by the European Medicines Agency in 2012, also has modest clinical activity in relapsed/refractory DLBCL [14,15]. Historical data of patients with refractory DLBCL treated with the best therapies before the availability of novel treatments showed dismal outcomes with an objective response rate (ORR) and a complete response (CR) rate of 26 and 7%, respectively, and median overall survival (OS) of only 6 months [16]. 

In 2017, the first chimeric antigen receptor (CAR) T-cell product was approved by the U.S. Food and Drug Administration (FDA) for the treatment of patients with relapsed/refractory DLBCL. With three products currently approved in the U.S, CAR T cells have transformed the treatment paradigm of DLBCL by providing a highly efficacious treatment option for patients with chemoresistant DLBCL who are ineligible for AHCT and for those whose lymphoma progresses or relapses after AHCT. However, despite the high response rates with CAR T cells in relapsed/refractory DLBCL, only 30–40% of patients achieve durable remissions [17,18,19,20,21]. In addition, treatment with CAR T cells results in significant toxicities, namely, cytokine-release syndrome (CRS, grade ≥ 3 reported in 2–22% of patients) and immune effector cell-associated neurotoxicity syndrome (ICANS, grade ≥ 3 reported in 10–31% of patients), which limit its use in older and unfit patients [17,18,19,21,22,23]. Further, the highly specialized and resource-intense requirements for treatment with CAR T cells, the long turnaround time between leukapheresis and the availability of the CAR T-cell product for infusion, and the inadequate bridging therapy options for patients with rapidly progressing disease are other important limitations of CAR T cells. 

Since 2019, the following four non-CAR T cell treatments have been approved by the FDA for patients with relapsed/refractory DLBCL (Table 1): polatuzumab vedotin in combination with BR (BR–pola) (June 2019), selinexor (June 2020), tafasitamab plus lenalidomide (July 2020), and loncastuximab tesirine (April 2021). These approvals provided new treatment options for the patients who are ineligible for AHCT and CAR T cells due to age/comorbidities and for those whose lymphoma progressed or relapsed after AHCT and CAR T cells. They have also been used as bridging therapies to AHCT and CAR T cells in patients with chemorefractory DLBCL. In this article, I review these new non-CAR T cell therapies and their roles in the treatment paradigm of relapsed/refractory DLBCL. I also discuss the novel agents currently under investigation that have shown promising early results in relapsed/refractory DLBCL.

## 2. FDA-Approved Therapies 

**Figure 1:**
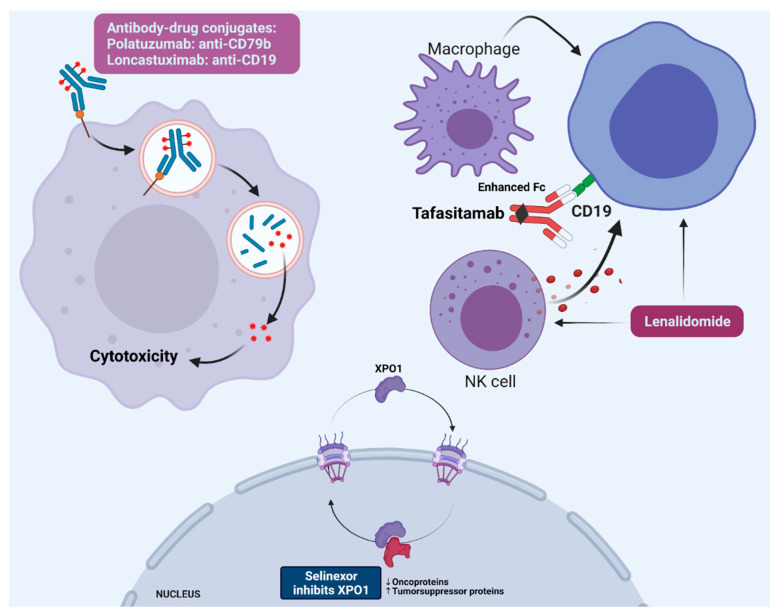
Mechanisms of action for recently approved novel therapies in relapsed/refractory DLBCL. Created with BioRender.com (accessed on 1 October 2021).

Table 1 summarizes the outcome and safety data for the four clinical trials that led to the approvals of BR–pola, selinexor, tafasitamab plus lenalidomide, and loncastuximab. Figure 1 illustrates their mechanisms of action. Caution should be exercised when comparing results across these clinical trials, especially given the relatively small number of patients in each trial, the heterogeneity of the enrolled patient populations, the differences in trial methodologies and follow-up periods, and the lack of direct comparisons.

### 2.1. BR–Pola

Polatuzumab is an antibody–drug conjugate comprising a humanized anti-CD79b monoclonal antibody covalently attached via a protease-cleavable linker to monomethyl auristatin E (MMAE). MMAE, a microtubule-disrupting antimitotic agent, is released once the polatuzumab/CD79b complex is internalized, and the linker cleaved. Early clinical trials showed that single agent polatuzumab had modest clinical activity in relapsed/refractory DLBCL and needed to be combined with other active agents [28,29]. 

In the GO29365 phase Ib/II clinical trial, polatuzumab was combined with bendamustine plus either rituximab (BR–pola) or obinutuzumab (BG–pola) in patients with relapsed/refractory DLBCL [12]. The treatment consisted of six 21-day cycles of BR–pola or BG–pola. The notable inclusion criteria were the receipt of ≥1 prior therapy, baseline peripheral neuropathy ≤ grade one, and ineligibility for AHCT. Patients with transformed follicular lymphoma (FL) were not included. Although patients with double- and triple-hit lymphomas were allowed, none were included. Focusing on the 80 patients randomized to BR alone or BR–pola, the median number of prior therapies was two, with 45 and 48% of the BR and BR–pola cohorts receiving ≥3 prior therapies, respectively. Most patients were refractory to their last prior therapy (BR 85%, BR–pola 75%). Patients were ineligible for AHCT most commonly due to age (BR 48%, BR–pola 33%) and an inadequate response to salvage therapy (BR 23%, BR–pola 30%). In addition, 25% of the patients in the BR–pola cohort and 15% in the BR cohort underwent prior AHCT. BR–pola resulted in a higher end-of-treatment CR rate (the primary endpoint: 40% vs. 18%), end-of-treatment ORR (45% vs. 18%), best ORR (63% vs. 25%), best CR rate (50% vs. 23%), and superior median progression-free survival (PFS) (10 vs. 4 months), duration of response (DOR) (13 vs. 8 months), and OS (12 vs. 5 months). Acknowledging the small sample size, the OS benefit with BR–pola was seen irrespective of age, refractoriness to last therapy, number of prior therapies, prior AHCT, DLBCL subtype based on COO by GEP, or MYC/BCL2 double-expression status. In patients treated with BR–pola with available data on COO (*n* = 32), the end-of-treatment ORRs seemed higher in the patients with ABC vs. GCB (59% vs. 33%), although the small sample size limits any definitive conclusions. The updated data presented in abstract form showed that a subset of patients (25%, *n* = 10) treated with BR–pola achieved durable remissions with ongoing responses lasting more than 25 months (range 26–49 months) [30]. Forty-four percent of the patients treated with BR–pola developed peripheral neuropathy (28% grade one, 15% grade two), which resolved in most patients (59%). Grade 3–4 neutropenia, thrombocytopenia, and anemia were more common with BR–pola (46, 41, and 28% vs. 33, 23, and 18%, respectively), but without an increased risk of neutropenic fever (BR–pola 10%, BR 13%) [12]. Based on these results, the FDA approved BR–pola for patients with relapsed/refractory DLBCL after at least two prior therapies. The POLARGO (NCT04182204) trial is an ongoing phase III study randomizing patients with relapsed/refractory DLBCL to R-GemOx alone or in combination with polatuzumab (Table 2). Polatuzumab is also being evaluated in combination with lenalidomide plus rituximab (NCT02600897) in relapsed/refractory DLBCL.

### 2.2. Selinexor 

Selinexor is a first-in-class oral selective XPO1 inhibitor. XPO1 (exportin 1) is a nucleo-cytoplasmic shuttling protein that plays an important role in exporting proteins from the nucleus to the cytoplasm and is overexpressed in DLBCL [31]. Inhibition of XPO1 results in nuclear accumulation, the activation of tumor suppressor proteins such as p53 and p21, and a reduction in oncoproteins such as c-Myc, Bcl2, and Bcl-X_L_ [24]. The phase II SADAL trial treated patients with relapsed/refractory DLBCL with selinexor in two dose groups, 60 mg and 100 mg twice weekly, until disease progression or intolerance [24]. The 100-milligram dose was discontinued as the 60-milligram dose resulted in a similar ORR and had a better safety profile. The notable inclusion criteria were the receipt of 2–5 prior lines of therapy, ineligibility for AHCT, and at least 60 days since last prior treatment for patients who responded to last prior treatment and at least 98 days for patients who did not respond. A total of 127 patients treated with the 60-milligram dose were included in the outcome and safety analyses. Twenty-four percent of the patients had DLBCL transformed from indolent lymphoma and 4% had double- or triple-hit lymphoma. The median number of prior therapies was two with 41% of patients receiving ≥4 prior therapies. Most patients (72%) were refractory to their last prior therapy and 30% underwent a prior AHCT. The median time from the last progression to starting selinexor was 8 weeks (range 5–15). Selinexor resulted in an ORR of 28%, including a CR in 12%. With a median follow-up of 15 months, the median PFS, DOR, and OS were 3, 9, and 9 months, respectively. The ORR was higher in the DLBCL GCB subtype (*n* = 59) by IHC than in non-GCB (*n* = 63) (34% vs. 21%), and in patients with low (<40%) c-Myc expression by IHC (42% vs. 13%). The notable adverse events of selinexor included grade 3–4 thrombocytopenia (46%), neutropenia (25%), and anemia (22%), nausea (all grades, 58%; grade three, 6%), vomiting (all grades, 30%), and hyponatremia (grade three, 8%) [24]. Based on these data, selinexor was approved by the FDA in patients with DLBCL after at least two prior systemic therapies. Several ongoing or planned clinical trials are evaluating selinexor in combination with chemotherapy (NCT02471911, NCT02741388) and novel agents such as ibrutinib (NCT02303392) and venetoclax (NCT03955783) in relapsed/refractory DLBCL (Table 2).

### 2.3. Tafasitamab plus Lenalidomide 

Tafasitamab is a humanized anti-CD19 monoclonal antibody engineered to enhance its binding to Fcγ receptors on immune cells and thus improve its antibody-dependent cell-mediated cytotoxicity and antibody-dependent cellular phagocytosis [32]. Although tafasitamab had limited clinical activity as monotherapy in relapsed/refractory B-cell non-Hodgkin lymphoma (NHL), it had remarkable clinical activity when combined with lenalidomide [33]. Lenalidomide has several mechanisms of action in lymphoid malignancies including immunomodulatory effects and direct antineoplastic and antiangiogenic activities [34]. Importantly, lenalidomide has shown synergism when combined with anti-CD20 monoclonal antibodies such as rituximab, as it enhances the NK-cell and antibody-dependent cell-mediated cytotoxicity of the anti-CD20 monoclonal antibody [35,36]. However, lenalidomide, with or without rituximab, showed limited clinical activity in relapsed/refractory DLBCL with an ORR of 19–28% and a median PFS of 3–4 months [11,37,38]. 

The phase II L-MIND trial evaluated the combination of tafasitamab and lenalidomide in patients with relapsed/refractory DLBCL ineligible for AHCT [25]. Patients who received 1–3 prior therapies were eligible. The notable exclusion criteria were double- and triple-hit lymphomas, primary refractory DLBCL (initially defined as no response or progression within 3 months of first-line therapy but later expanded to within 6 months of first-line therapy), and previous treatment with anti-CD19 therapies. The patients received treatment with lenalidomide plus tafasitamab for up to twelve 28-day cycles, followed by tafasitamab monotherapy in patients with stable disease or better until disease progression. Eighty-one patients were enrolled and 80 were evaluable for efficacy. The median number of prior therapies was two, with 50% of patients receiving one prior therapy and only 7% receiving ≥3. Forty-four percent of patients had disease refractory to the last prior therapy and 11% underwent a prior AHCT. Patients were ineligible for AHCT most commonly due to age (46%), chemorefractory disease (23%), refusal (14%), or comorbidities (14%). Nine percent had DLBCL transformed from indolent lymphoma. The best ORR was 58% including a CR in 40%. The median DOR was 44 months and was not reached in patients who achieved a CR. The median PFS and OS were 12 and 34 months, respectively [26]. The ORR was not significantly different among the patients with primary refractory disease (*n* = 15, ORR 53%) or those with disease refractory to their last therapy (*n* = 35, ORR 60%), but the median PFS (5 and 8 months, respectively) and OS (14 and 16 months, respectively) were shorter in both cohorts compared with the overall trial population. The median PFS and OS were also significantly shorter in the patients who received ≥2 prior therapies (8 and 15 months, respectively) compared with those who received one prior therapy (24 and 46 months, respectively). Based on the COO by IHC, the ORR was higher in the patients with non-GCB (*n* = 22, ORR = 68%) vs. GCB (*n* = 38, ORR = 48%) [39]. The main toxicities were grade ≥ 3 neutropenia (48%) and thrombocytopenia (17%), febrile neutropenia (12%), rash (36%; grade three, 9%), and diarrhea (33%; grade three, 1%). Based on the L-MIND study results, the FDA approved the combination of tafasitamab and lenalidomide for patients with relapsed/refractory DLBCL. Tafasitamab is being evaluated in combination with other active agents in several trials including a randomized phase II/III trial in combination with bendamustine compared with BR in relapsed/refractory DLBCL (B-MIND, NCT02763319). Lenalidomide is being evaluated in combination with multiple novel agents, as discussed throughout the text and shown in Table 2. 

### 2.4. Loncastuximab Tesirine 

Loncastuximab is an anti-CD19 humanized monoclonal antibody conjugated to a pyrrolobenzodiazepine dimer, a payload that results in interstrand DNA crosslinking. The LOTIS-2 phase II trial evaluated loncastuximab in 145 patients with relapsed/refractory DLBCL [27]. The notable inclusion criteria were prior treatment with ≥2 systemic therapies and the presence of biopsy-proven CD19 expression for patients with prior CD19-directed treatments. Loncastuximab was administered every 3 weeks for up to one year with an option of continuing treatment beyond one year for those patients deriving a clinical benefit. The median number of prior therapies was three, with 56% of the patients receiving ≥3 prior therapies. Fifty-eight percent had disease refractory to their last prior treatment, 14% underwent a prior AHCT, and 9% received prior CAR T-cell therapy. Twenty percent had DLBCL transformed from indolent lymphoma. Loncastuximab resulted in an ORR of 48%, including a CR in 24%. Of the 15 patients with double- or triple-hit lymphoma, five patients responded (33%, all a CR). The median DOR was 10 months and for the patients with a CR, it was 13 months. The median PFS and OS were 5 and 10 months, respectively. The ORRs were similar, irrespective of the number of prior lines of therapy (2, 3, or ≥4; 48–49%) and in transformed vs. de novo DLBCL (45% vs. 49%) but was lower in the patients with disease refractory to their last prior therapy vs. not (37% vs. 67%). Based on the COO by GEP, the ORR was not significantly different in GCB vs. ABC (54% vs. 48%, respectively). Of the 13 patients who received prior treatment with CAR T cells, six (46%) responded (two with a CR, 15%), and of the 15 patients who received CAR T-cell therapy after loncastuximab, seven (47%) responded (six with a CR, 40%). The notable adverse events seen with loncastuximab include grade ≥ 3 neutropenia (26%) and thrombocytopenia (18%), grade ≥3 increased gamma-glutamyltransferase (17%), edema/effusion (all grade 31%; grade ≥3, 5% including three patients with pleural and two with pericardial effusions), and skin/nails adverse events (rash, erythema, photosensitivity reactions) (all grade 43%; grade ≥3, 4%) [27]. Based on these data, the FDA approved loncastuximab in patients with relapsed/refractory DLBCL after two or more lines of systemic therapy. Loncastuximab is being evaluated in combination with ibrutinib (NCT03684694), venetoclax (NCT05053659), and in a phase III trial in combination with rituximab versus R-GemOx in patients with relapsed/refractory DLBCL (NCT04384484) (Table 2). 

### 2.5. Clinical Considerations

The addition of BR–pola, selinexor, tafasitamab plus lenalidomide, and loncastuximab to the treatment armamentarium for patients with relapsed/refractory DLBCL who are ineligible for AHCT or CAR T-cells is certainly needed and welcomed. The efficacy of these new approvals in patients whose lymphoma progressed or relapsed after AHCT or CAR T cells remain undefined as only a minority of patients treated with these agents on clinical trials had undergone AHCT (11–30%) and/or CAR T cells (0–9%) [12,24,25,27]. Further, the data on the role of these treatments as bridging therapies to AHCT or CAR T cells are limited. The optimal sequence of these treatments also remains unclear. The FDA approvals of selinexor, tafasitamab plus lenalidomide, and loncastuximab were based on single-arm phase I/II clinical trials, whereas BR, the comparator in the BR–pola trial, has modest clinical activity. By acknowledging the absence of direct comparisons, lack of reliable predictive biomarkers, and the caveats of cross-trial comparisons, several important points can be taken into consideration when selecting among these treatments for patients with relapsed/refractory DLBCL ineligible for AHCT and CAR T cells (Table 1). Overall, the data support the use of BR–pola, tafasitamab plus lenalidomide, and loncastuximab before selinexor for most patients. Selinexor resulted in the lowest response rate (ORR 28%, CR rate 12%) and shortest median PFS (3 months) [24]. In addition, the requirement for a “washout” period of 60 or 98 days (depending on the response to last prior therapy) on the SADAL trial is concerning for selection bias by which patients with the less aggressive disease were favorably enrolled on trial. However, the SADAL trial still included high-risk patient populations (such as 72% with refractory to their last prior treatment and 30% with a prior AHCT) [40]. Selinexor has the advantage of being an oral agent, but it still has considerable toxicities, including high rates of myelosuppression, nausea/vomiting, constitutional symptoms, and grade three hyponatremia. Tafasitamab plus lenalidomide resulted in high response rates and has the most data thus far on response durability (median DOR of 44 months) compared with BR–pola and loncastuximab [26]. However, compared with patients treated on the GO29365 and LOTIS-2 trials, those treated with tafasitamab plus lenalidomide on the L-MIND trial were less heavily pretreated (50% with one prior treatment vs. 0–28%, 7% with ≥3 prior treatments vs. 45–56%) and were less likely to have disease refractory to their last prior treatment (44% vs. 58–75%). The LOTIS-2 trial of loncastuximab had more patients with double- and triple-hit lymphoma (*n* = 15, 10%) and prior treatment with CAR T cells (*n* = 13, 9%), although the small numbers in each group hinder a reliable evaluation of loncastuximab’s efficacy in these very high-risk and difficult-to-treat patients. Time-limited treatment with BR–pola is advantageous over treatment until progression with selinexor and tafasitamab plus lenalidomide (although treatment with lenalidomide is limited to the first year) and for at least one year with loncastuximab. Treatment with BR–pola might be limited by pre-existing peripheral neuropathy, a common problem in patients with DLBCL with prior treatment with vinca alkaloids and/or platinum agents. Bendamustine can cause the prolonged suppression of T cells and, therefore, may be better avoided before CAR T-cell collection if used as a bridging therapy [41,42]. Unlike tafasitamab, loncastuximab, and the currently approved CAR T cell products, which target CD19, polatuzumab targets CD79b and, therefore, treatment with BR–pola might be preferred in patients who progress on prior CD19-directed therapies. Notably, the limited data show that progression on one type of CD19-directed therapy does not preclude responses to other CD19-directed therapies as evident by the responses seen in the small number of patients who received CAR T cells before loncastuximab on the LOTIS-2 trial (although biopsy-proven CD19 expression was required in the LOTIS-2 trial) as well as responses to CAR T-cell therapies in patients who received prior loncastuximab or lenalidomide plus tafasitamab [27,43,44]. 

## 3. Emerging Therapies

### 3.1. Bispecific Antibodies

The bispecific antibodies comprise two unique single-chain variable fragments connected via small linker peptides with one fragment designed to bind to CD3 on T cells and the other to a tumor-associated antigen. The simultaneous binding of the tumor-associated antigen and CD3 induces T-cell mediated cytotoxicity of the target cell. Blinatumomab, which binds to CD3 and CD19, had limited activity in B-cell NHL [45]. However, several bispecific antibodies targeting CD3 and CD20 have shown very encouraging early results in relapsed/refractory DLBCL (Table 3). Being a type of T-cell-redirecting therapy, the bispecific antibodies share several features in common with CAR T-cells, including the risk of CRS and ICANS. However, the rate and severity of CRS and ICANS associated with the bispecific antibodies have been successfully reduced by the implementation of several mitigation strategies, including pre-medications with steroids and/or anti-CD20 monoclonal antibodies, split and step-up dosing, and subcutaneous formulations (which result in lower peak cytokine levels). Unlike the currently approved CAR T cells, the bispecific antibodies are readily available “off-the-shelf” and do not require a manufacturing process individualized for each patient or lymphodepleting chemotherapy. However, whereas CAR T cells are typically infused once, repetitive dosing with the bispecific antibodies is needed but the optimal duration of treatment is still unclear. Importantly, despite the high response rates seen with the bispecific antibodies, data on their response durability are still limited. 

Glofitamab is an IgG1 fully humanized anti-CD20xCD3 bispecific antibody composed of two binding sites for CD20 and one for CD3 [46]. Glofitamab’s CD20 bivalency allows it to be combined with competing anti-CD20 monoclonal antibodies such as obinutuzumab [52]. NP30179 is an ongoing phase I trial that recently reported on the safety and preliminary efficacy of glofitamab in patients with relapsed/refractory DLBCL, other related aggressive B-cell NHL, or FL [46]. The patients received one dose of obinutuzumab seven days before glofitamab for B-cell depletion and CRS mitigation. In total, 171 patients were included, 127 with aggressive B-cell NHL including 73 with DLBCL, 29 with transformed FL, and 44 with FL. The patients received a median of three prior therapies. Twenty-four percent underwent a prior AHCT and only 2% received prior CAR T cells. The ORR was 54% including a CR in 37. In the 35 patients treated at the recommended phase II dose, the ORR was 66%, including a CR in 57%. The ORR and CR rates were 41 and 29% in DLBCL and 55 and 35% in transformed FL, respectively. With a median follow-up of 14 months, the median PFS was 3 months in aggressive NHL and 12 months in FL. Although the follow-up is still short, the responses seemed durable in patients with a CR, with 73% of patients with aggressive NHL maintaining their remission at 12 months. CRS occurred in 50% of all the patients, including grades 3–4 in 4% and was more common during the first treatment cycle (13% with cycle two and 6% with cycle three or later). Neurological adverse events occurred in 43% of patients with ICANS-like adverse events in 5% (none grade ≥ 3). Only 3% of the patients discontinued treatment due to adverse events [46]. A phase III trial (NCT04408638) is evaluating the combination of glofitamab, gemcitabine, and oxaliplatin vs. R-GemOx in relapsed/refractory DLBCL. Glofitamab is also being evaluated in combination with obinutuzumab and RO7227166, a CD19x4-1BB bispecific antibody (NCT04077723), and in combination with polatuzumab or atezolizumab (programmed cell death-1 ligand 1 [PD-L1] inhibitor) (NCT03533283) (Table 2).

Epcoritamab is an IgG1 bispecific antibody derived from a human anti-CD20 monoclonal antibody and a humanized anti-CD3 monoclonal antibody using DuoBody technology, a platform that allows the production of bispecific antibodies by exchanging half-molecules from different parental IgGs [47,53]. In its ongoing first-in-human phase I/II trial, 68 patients with relapsed/refractory B-cell NHL were treated including 46 with DLBCL. The patients received epcoritamab subcutaneously in escalating doses ranging from 0·0128 to 60 mg. The patients received a median of three prior therapies including AHCT in 15% and CAR T cells in 11%. Eighty-nine percent of the patients were refractory to their last prior therapy. In the 22 patients with DLBCL who received epcoritamab at doses of ≥12 mg, the ORR was 68%, including a CR in 45%. The median PFS for patients with DLBCL treated at doses of ≥12 mg was 9 months. All four evaluable patients with DLBCL who received prior CAR T cells responded, including two who achieved a CR. CRS occurred in 59% of the patients without any grade ≥3 events and with most events occurring during the first treatment cycle. Neurological symptoms occurred in four patients (8%) (ICANS grading was not used). No patients discontinued epcoritamab due to adverse events [47]. An ongoing phase III trial is evaluating epcoritamab vs. investigator’s choice chemotherapy in relapsed/refractory DLBCL. Epcoritamab is also being evaluated in combination with R-GemOx in AHCT-ineligible patients or with R-DHAX/C (rituximab, cytarabine, dexamethasone, and oxaliplatin/carboplatin) in AHCT-eligible patients with relapsed/refractory DLBCL (NCT04663347) (Table 2).

Mosunetuzumab is a humanized IgG1 anti-CD20xCD3 bispecific antibody that has also shown promising activity in relapsed/refractory DLBCL. In the phase I/Ib GO29781 trial presented in abstract form, 270 patients with B-cell NHLs, including 180 patients with aggressive NHLs (DLBCL *n* = 117, transformed FL *n* = 32), were treated with mosunetuzumab intravenously [48]. The patients received a median of three prior therapies and 72% were refractory to their last prior treatment. Thirty patients (11%), including 25 patients with DLBCL or transformed FL, received prior CAR T cell therapy. CRS occurred in 29% of the patients (grade three in 1%), whereas ICANS-like events occurred in 1%. In patients with aggressive NHLs, the ORR was 37%, including a CR in 19%, with responses seen in patients who received prior CAR T-cell therapy (18 patients evaluable, an ORR in 39% including a CR in 22%) [48]. Mosunetuzumab was also evaluated as a subcutaneous injection in 46 patients with B-cell NHLs and showed similar safety (CRS 28%, no grade ≥ 3) and efficacy data (ORR of 44% including a CR in 22% in 36 patients with aggressive NHLs) [49]. Mosunetuzumab is being evaluated in combination with other agents including polatuzumab (NCT03671018) and atezolizumab (NCT02500407) and with chemotherapy (GemOx, NCT04313608) (Table 2).

Odronextamab is a fully human IgG4 anti-CD20xCD3 bispecific antibody that was evaluated in a phase I study of 136 patients with relapsed/refractory B-cell NHL including 78 patients with DLBCL [50]. Based on data presented in abstract form, the patients received a median of three prior therapies, and 80% were refractory to their last prior treatment. The ORR was 55% (all CRs) in the 11 evaluable patients with DLBCL who did not receive prior CAR T cells, and 33% including a CR in 21% of the 24 patients with DLBCL and prior treatment with CAR T cells. The median DOR was not reached in both groups. In the patients with DLBCL, CRS occurred in 63% of patients, including grade three in 5% and grade ≥3 ICANS-like adverse events occurred in 4% of the patients. The median DOR for both groups was not reached [50]. 

Plamotamab is a humanized IgG1 anti-CD20xCD3 bispecific antibody that was evaluated in a phase I trial of patients with relapsed/refractory B-cell NHL or chronic lymphocytic leukemia [51]. The data presented in abstract form showed an ORR of 50% including a CR in 28% of the 18 patients with DLBCL. CRS occurred in 53% of the patients, including grades 3–4 in 6%. Neurological events occurred in 49% of the patients with dizziness (17%), headache (17%), paresthesia (9%), and lethargy (6%) being the most common [51].

### 3.2. Antibody–Drug Conjugates 

In addition to polatuzumab and loncastuximab, several antibody–drug conjugates are being evaluated in DLBCL. Naratuximab is an anti-CD37 monoclonal antibody conjugated to the maytansinoid derivative, DM1. In a phase I trial of 49 patients with relapsed/refractory B-cell NHL, naratuximab resulted in an ORR of 22% in the 18 patients with DLBCL (including a CR in one patient (6%)) [54]. A phase II study evaluated naratuximab in combination with rituximab in 100 patients with relapsed/refractory B-cell NHL, including 80 with DLBCL [55]. The most common grade ≥ 3 treatment-emergent adverse events were neutropenia (54%), lymphopenia (17%), and thrombocytopenia (11%). In the 74 evaluable patients with DLBCL, the ORR and CR rates were 43 and 32%, respectively [55]. Zilovertamab vedotin is an MMAE-conjugated humanized IgG1 monoclonal antibody that targets ROR1, an oncofetal protein pathologically expressed in solid and lymphoid malignancies including DLBCL [56]. A phase I study evaluated zilovertamab in 32 patients with relapsed/refractory B-cell NHLs [57]. Three out of the five patients with relapsed/refractory DLBCL responded and two achieved a CR. Responses were also seen in 7 out of the 15 patients with mantle cell lymphoma (MCL). The toxicities were mainly neutropenia (grade ≥ 3, 34%) and peripheral neuropathy (44%; grade three, 13%) [57]. TRPH-222, an antibody–drug conjugate targeting CD22, resulted in limited activity in DLBCL in a phase I trial that included 10 patients with DLBCL/transformed FL with an ORR of 20% [58]. 

### 3.3. Bruton Tyrosine Kinase (BTK) Inhibitors

Despite their transformative role in the treatment landscape of several B-cell lymphoid malignancies, the BTK inhibitors have limited single-agent activity in DLBCL. Small phase I/II trials show preferential activity for the BTK inhibitors in the ABC/non-GCB subtype of DLBCL with an ORR of 24–37% but a short DOR (4–8 months) [10,59,60]. However, the BTK inhibitors might have a role in DLBCL when used in combination with other active agents. A phase Ib trial evaluated the combination of ibrutinib, lenalidomide, and rituximab in 45 patients with relapsed/refractory DLBCL who are ineligible for AHCT [61]. The ORR and CR rates were 44 and 28%, and 65 and 41% in the 17 patients with non-GCB DLBCL, respectively. The median DOR, PFS, and OS were 16, 6, and 10 months for the overall cohort, and 16, 4, and 11 months for the patients with non-GCB DLBCL, respectively. In a phase II of the same combination in 89 patients with non-GCB DLBCL presented in abstract form, the ORR and CR rates were 47 and 28%, respectively [62]. The median DOR, PFS, and OS were 18, 5, and 14 months, respectively. The BTK inhibitors are being evaluated in combination with other agents in several trials as shown in Table 2 and discussed throughout the text. 

### 3.4. BCL2 Inhibitors

Venetoclax is an oral selective inhibitor of BCL-2, an important anti-apoptotic molecule overexpressed in DLBCL [63]. In a phase I clinical trial of patients with relapsed/refractory B-cell NHL, venetoclax showed modest clinical activity in the 34 patients with DLBCL (ORR = 18%, CR = 12%) [64]. A phase Ib/II evaluated the combination of venetoclax, ibrutinib, prednisone, obinutuzumab, and lenalidomide (VIPOR) in 58 patients with relapsed/refractory B-cell NHL [65]. In a preliminary analysis presented in abstract form, VIPOR resulted in an ORR of 55% including a CR in 35% of the 31 patients who had DLBCL or double-/triple-hit lymphoma and were evaluable for response. The ORR was 64%, including a CR in 57% of the 14 patients with non-GCB DLBCL with a 1-year PFS rate of 44%. Venetoclax is also being evaluated in combination with polatuzumab and rituximab in a phase Ib/II in patients with relapsed/refractory DLBCL [66]. In a preliminary analysis presented in abstract form, the combination resulted in an ORR of 65% including a CR in 38% of the 48 patients evaluable for response. With a median follow-up of 7 months, the median DOR, PFS, and OS were 6, 4, and 11 months, respectively. Ongoing clinical trials are evaluating venetoclax in relapsed/refractory DLBCL in combination with other agents, including rituximab plus ibrutinib (NCT03136497), obinutuzumab plus lenalidomide (NCT02992522), and R-ICE chemotherapy (rituximab, ifosfamide, carboplatin, etoposide; NCT03064867) (Table 2).

### 3.5. Immune Checkpoint Inhibitors

Programmed cell death protein 1 (PD-1) inhibitors have shown disappointing results in relapsed/refractory DLBCL with an ORR of 3–10% with nivolumab monotherapy [67]. However, retrospective data suggest that the checkpoint inhibitors might sensitize lymphoma to subsequent treatments with chemotherapy or novel agents [68]. PD-1 and PD-L1 inhibitors are being evaluated in combination with other novel agents including the bispecific antibodies (Table 2). The macrophage immune checkpoint inhibitor, magrolimab, is a humanized monoclonal antibody against CD47, a cell surface receptor involved in inhibiting tumor-cell phagocytosis by macrophages and dendritic cells [69]. Upon binding to signal regulatory protein a (SIRPa) on phagocytic cells, CD47 provides what is known as a “do not eat me” signal. By blocking CD47, magrolimab induces tumor phagocytosis. In a phase I trial of 22 patients with relapsed/refractory DLBCL or FL, most of whom were refractory to rituximab, magrolimab plus rituximab resulted in ORR and CR rates of 50 and 36%, respectively [70]. Infusion-related reactions and anemia were the main treatment toxicities. A phase II study of magrolimab in combination with R-GemOx or rituximab alone is ongoing (NCT02953509) (Table 2). SRF231, an anti-CD47 monoclonal antibody, and TTI-621, a CD47 decoy receptor, are other agents targeting CD47/SIRPa being evaluated in clinical trials (NCT03512340 and NCT02663518, respectively).

### 3.6. Epigenetic Modifiers

EZH2, a histone methyltransferase that has a key role in germinal center formation, regulates B cell differentiation and promotes cell proliferation [71]. Recurrent gain-of-function mutations in *EZH2* occur in up to 22% of GCB DLBCL and 28% of FL [72,73,74,75]. Tazemetostat is an EZH2 inhibitor approved for the treatment of patients with relapsed FL. However, its clinical activity in relapsed/refractory DLBCL is modest, irrespective of *EZH2* mutation status, with an ORR of 17% [76]. Valemetostat, an EZH1/2 dual inhibitor, is being evaluated in relapsed/refractory NHL, including DLBCL (NCT04842877). 

Histone acetylation is a key epigenetic regulator of gene expression and plays an important role in lymphomas, including DLBCL. Loss-of-function mutations in genes encoding proteins involved in histone acetylation such as *EP300* and *CREBBP* are found in 25% of patients with DLBCL [77]. Although the histone deacetylase (HDAC) inhibitors have established therapeutic roles in T-cell lymphomas and multiple myeloma, their activity in DLBCL is limited. Abexinostat, an oral pan-HDAC inhibitor, showed an ORR of 31% (CR in 6%) in 17 patients with relapsed/refractory DLBCL treated as part of a phase II trial of 100 patients with various types of NHL. The median DOR was only 2 months in patients with DLBCL [78]. Abexinostat is being evaluated in combination with ibrutinib in patients with relapsed/refractory DLBCL or MCL (NCT03939182). Similar results were seen with another oral pan-HDAC inhibitor, panobinostat, which resulted in an ORR of 28% (CR in 18%) when given alone or in combination with rituximab in 40 patients with relapsed/refractory DLBCL [79]. Other HDAC inhibitors such as vorinostat, belinostat, and mocetinostat had disappointing results as monotherapies in relapsed/refractory DLBCL [80,81,82]. 

Bromodomain and extra-terminal (BET) proteins act as “readers” of histone acetylation and are involved in regulating gene transcription. They are implicated in the development and progression of various malignancies, including B-cell NHL, where they might activate the *MYC* and *BCL2* signaling pathways [83,84]. BET inhibitors used as monotherapy (birabresib, CC-90010, INCB054329, INCB057643, CPI-0610, RO6870810) had disappointing results in DLBCL (ORR 0–14%) [85,86,87,88,89]. However, a phase Ib study showed promising results for RO6870810 in combination with venetoclax and rituximab in 39 patients with relapsed/refractory DLBCL [90]. The ORR was 39%, including a CR in 21%, with 48% of responses lasting ≥6 months. The most common grade ≥ 3 adverse events were neutropenia (28%), anemia (23%), thrombocytopenia (23%), and neutropenic fever (10%). 

Protein arginine methyltransferases (PRMTs) catalyze the arginine methylation of histones as a posttranslational modification resulting in gene silencing [84]. PRMT5 is upregulated in various malignancies including lymphoma with preclinical data supporting the use of PRMT5 inhibitors in DLBCL and other B-cell NHLs [91,92]. The PRMT5 inhibitors, GSK3326595 and JNJ-64619178, are being evaluated in clinical trials in relapsed/refractory B-cell NHL including DLBCL (NCT02783300 and NCT03573310, respectively). 

## 4. Conclusions

These newly approved agents provide more treatment options for patients with relapsed/refractory DLBCL and reflect the remarkable progress made in the treatment landscape of DLBCL. However, we need to identify clinical and molecular biomarkers to better select treatments for individual patients. Multiple exciting new treatments are also on the horizon, with strong preliminary data for the bispecific antibodies in particular. Rational combinations of these novel agents will likely be required to improve response rates and achieve durable remissions.

## Figures and Tables

**Table 1:** Summary of clinical trials of BR–polatuzumab, selinexor, tafasitamab plus lenalidomide, and loncastuximab.

Variable	BR–Polatuzumab	Selinexor	Tafasitamab and Lenalidomide	Loncastuximab
Trial [references]	GO29365 [12] *	SADAL [24]	L-MIND [25,26]	LOTIS-2 [27]
No. of patients enrolled	40	127	81	145
Age, median (years)	67	67	72	66
Bulky disease, % (longest dimension cutoff)	25% (≥7.5 cm)	Not reported	19% (≥7.5 cm)	6% (≥10 cm)
Double-/triple-hit lymphoma, %	0	4%	3%	10%
No. of prior therapies, median (range)	2 (1–7)	2 (2–6)	2 (1–4)	3 (IQR 2–4)
≥3 prior therapies, %	45%	41% (≥4 therapies)	7%	56%
Primary refractory, %	Not reported	Not reported	19% **	20%
Refractory to last treatment, %	75%	72%	44%	58%
Prior AHCT, %	25%	30%	11%	14%
Prior CAR T, %	0%	0%	0%	9%
Best ORR, %	63%	28%	58%	48%
Best CR, %	50%	12%	40%	24%
Follow-up, median (months)	22	15	34	Not reported
DOR, median (months)	13	9	44	10
PFS, median (months)	10	3	12	5
OS, median (months)	12	9	34	10
Neutropenia, G ≥ 3, %	46%	25%	49%	26%
Thrombocytopenia, G ≥ 3, %	41%	46%	17%	18%
Neutropenic fever, %	10%	3%	12%	3%
Adverse events of interest	Peripheral neuropathy 44% (G1, 28%; G2, 15%)	Hyponatremia (G3, 8%), nausea 58% (G3, 6%), vomiting 30%	Pneumonia 22%, tumor flare 4%, diarrhea 36% (G 3, 1%)	↑GGT (G ≥ 3, 17%), edema/effusion 31% (G ≥ 3, 5%), rash 43% (G ≥ 3, 4%)

* Data are shown for the BR–polatuzumab cohort only. ** Patients with disease relapse or progression within 3 months of first-line therapy were initially excluded from the trial but this was later expanded to within 6 months of first-line therapy. Abbreviations: AHCT, autologous hematopoietic stem cell transplantation; CAR T, chimeric antigen receptor T cells; CR, complete response; DOR, duration of response; G, grade; ↑ GGT, increased gamma-glutamyltransferase; IQR, interquartile range; ORR, objective response rate; OS, overall survival; PFS, progression-free survival.

**Table 2:** Select ongoing or planned clinical trials of novel agents in relapsed/refractory DLBCL.

Agent/Combination	Phase	Identifier (Trial Name)
R-GemOx +/− polatuzumab	III	NCT04182204 (POLARGO)
Polatuzumab, lenalidomide, rituximab	Ib/II	NCT02600897
Selinexor plus R-ICE	I	NCT02471911
Selinexor plus R-DHAX or R-GDP	Ib	NCT02741388 (SELINDA)
Selinexor and ibrutinib	I	NCT02303392
Selinexor and venetoclax	Ib	NCT03955783
Tafasitamab plus bendamustine vs. BR	II/III	NCT02763319 (B-MIND)
Loncastuximab plus ibrutinib	I/II	NCT03684694
Loncastuximab plus venetoclax	I	NCT05053659
Loncastuximab plus rituximab vs. R-GemOx	III	NCT04384484 (LOTIS-5)
Glofitamab, RO7227166, and obinutuzumab	I/II	NCT04077723
Glofitamab or mosunetuzumab plus GemOx	Ib	NCT04313608
Glofitamab plus atezolizumab or polatuzumab	Ib/II	NCT03533283
Glofitamab plus GemOx vs. R-GemOx	III	NCT04408638 (STARGLO)
Epcoritamab vs. investigator’s choice chemotherapy	III	NCT04628494 (GCT3013-05)
Epcoritamab plus R-DHAX/C or R-GemOx	Ib/II	NCT04663347
Mosunetuzumab and polatuzumab	Ib/II	NCT03671018
Mosunetuzumab and atezolizumab	I/II	NCT02500407
Venetoclax, ibrutinib, and rituximab	I	NCT03136497
Venetoclax, lenalidomide, and obinutuzumab	I	NCT02992522
Venetoclax plus R-ICE	I/II	NCT03064867
Magrolimab plus rituximab or R-GemOx	Ib/II	NCT02953509
Abexinostat and ibrutinib	I	NCT03939182

Abbreviations: BR, bendamustine and rituximab; R-DHAX/C, rituximab, cytarabine, dexamethasone, and oxaliplatin/carboplatin; R-GDP, rituximab, gemcitabine, dexamethasone, and cisplatin; R-GemOx, rituximab, gemcitabine, and oxaliplatin; R-ICE, rituximab, ifosfamide, carboplatin, and etoposide.

**Table 3:** Summary of clinical trials of bispecific antibodies in relapsed/refractory DLBCL.

Variable	Glofitamab [46]	Epcoritamab [47]	Mosunetuzumab [48,49]	Odronextamab [50]	Plamotamab [51]
Antibody structure	IgG1 humanized with 2:1 format	IgG1 using DuoBody technology	Humanized IgG1	Fully human IgG4	Humanized IgG1
Route of administration	IV	SQ	IV, SQ	IV	IV
Patients enrolled, n	171	68	270	136	53
Patients with DLBCL, n	73	46	117	78	18
No. of prior treatments, median *	3	3	3	3	3
Refractory to last treatment *	Not reported	85%	72%	80%	Not reported
Prior AHCT *	24%	15%	29%	7%	Not reported
Prior CAR T *	2%	9%	11%	26%	Not reported
Patients with DLBCL evaluable for response, n	73	22	124 with aNHL (tFL, MCL and others)	35	18
ORR, CR rate	41%, 29%	68%, 45% (at doses ≥12 mg)	37%, 19% (in aNHL)	No prior CAR T (*n* = 11): 55%, 55% Prior CAR T (*n* = 24): 33%, 21%	39%, 28%
CRS, any grade (grade ≥ 3) *	50% (4%)	57% (0%)	29% (1%)	61% (7%)	53% (6%)
ICANS-like, any grade (grade ≥ 3) *	5% (0%)	Neurological symptoms 8% (4%)	1% (0%)	Not reported (2%)	Not reported

* Including for patients with non-DLBCL histologies. Abbreviations: AHCT, autologous hematopoietic stem cell transplantation; aNHL, aggressive B-cell non-Hodgkin lymphoma; CAR T, chimeric antigen receptor T cells; CR, complete response; CRS, cytokine release syndrome; ICANS, immune effector cell-associated neurotoxicity syndrome; IV, intravenously; MCL, mantle cell lymphoma; ORR, objective response rate; SQ, subcutaneously; tFL, transformed follicular lymphoma.
